# Ewing’s Sarcoma of Mandible: An Impressive Case of Spontaneous Mandible Regeneration

**DOI:** 10.5005/jp-journals-10005-1376

**Published:** 2016-09-27

**Authors:** Ioannis Chatzistefanou, Sotiria Kabesi, Konstantinos Paraskevopoulos, Dimitrios Koliouskas, Konstantinos Antoniades

**Affiliations:** 1 Resident, Department of Oral and Maxillofacial Surgery, Papanikolaou General Hospital, Aristotle University of Thessaloniki Thessaloniki, Greece; 2 Student, Dental School, Aristotle University of Thessaloniki, Thessaloniki, Greece; 3 Resident, Department of Oral and Maxillofacial Surgery, Papanikolaou General Hospital, Aristotle University of Thessaloniki Thessaloniki, Greece; 4 Consultant and Chief, Department of Pediatrics, Hippokration General Hospital of Thessaloniki, Thessaloniki, Greece; 5 Professor, Department of Oral and Maxillofacial Surgery, Papanikolaou General Hospital, Aristotle University of Thessaloniki Thessaloniki, Greece

**Keywords:** Mandibulectomy, Ewing’s Sarcoma, Neuroectodermal.

## Abstract

Ewing’s sarcoma (ES) is an uncommon aggressive bone malignancy that mainly affects children and adolescents. Mandible involvement is quite rare and usually represents metastasis from another skeletal site. Combined therapy including wide surgical resection and preoperative and postoperative chemotherapy has been demonstrated as the mainstay of therapeutic approach. As improved therapeutic modalities have significantly increased survival over the last decades, functional and esthetic reconstruction of the postmandibulectomy defect is usually necessary for patient’s total rehabilitation. We report a case of ES of the mandible with special consideration to the postresection spontaneous structural and functional regeneration of the mandible.

**How to cite this article:**

Chatzistefanou I, Kabesi S, Paraskevopoulos K, Koliouskas D, Antoniades K. Ewing’s Sarcoma of Mandible: An Impressive Case of Spontaneous Mandible Regeneration. Int J Clin Pediatr Dent 2016;9(3):273-277.

## INTRODUCTION

Ewing’s sarcoma (ES) is a poorly differentiated primary bone malignancy that mainly affects children and adolescents.^1-3^ Since its initial description by James Ewing in 1921,^4^ ES remains a neoplasm of unclear pathogenesis. More than 50% of ES cases originate in the pelvis and long bones,^2,5,6^ with distant dissemination at diagnosis being the rule rather than the exception, reflecting its aggressive biological behavior.^7^

Facial bones are rarely involved (1-2%) and when do so their involvement usually represents metastatic disease from a primary skeletal lesion.^8-11^ The mandibular ramus is the predominant site of occurrence, with only few cases reported in the anterior mandible or maxilla.^12-17^ As improved therapeutic modalities have significantly increased survival, the current maxillofacial surgeon may need to face the challenge of an extensive surgical resection followed by a demanding functional and esthetic reconstruction of the ablative defect. We report a case of an ES developed in mandibular ramus of a 2-year-old girl with special consideration to the postmandibulectomy spontaneous structural and functional bone regeneration that made any plans for secondary mandible reconstruction unnecessary.

## CASE REPORT

**Fig. 1:**
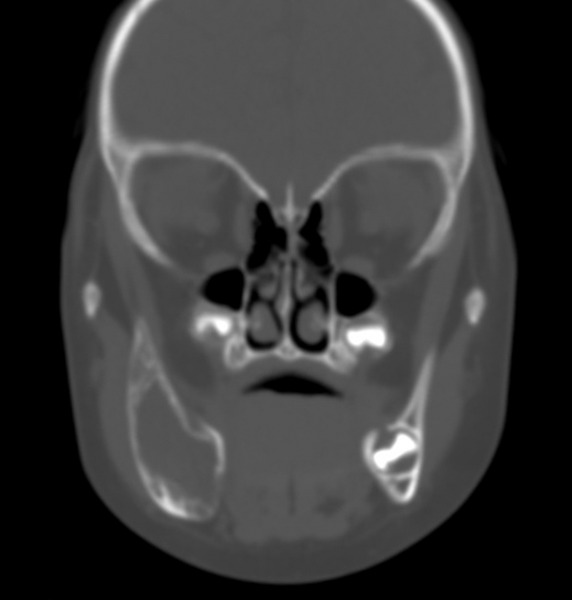
Computed tomography scan showing a suspicious expansive lytic lesion of the mandible with cortical erosion

**Fig. 2:**
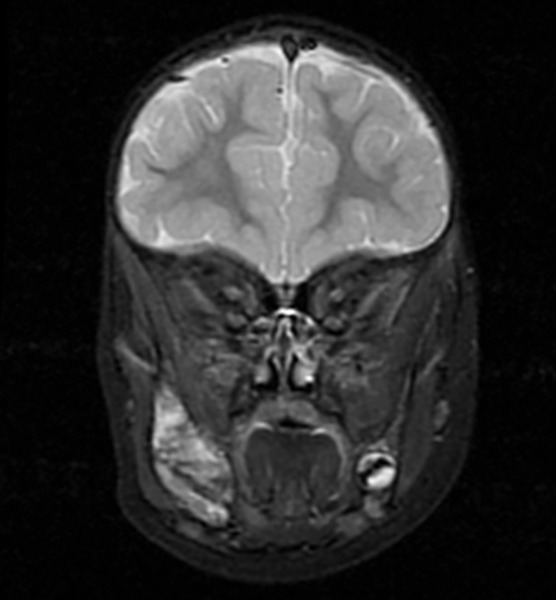
Magnetic resonance imaging scan showing soft tissue involvement

A 2-year-old girl with a 3-month history of a painless, progressively deteriorating swelling in the right mandible was referred to the Oral and Maxillofacial Department for evaluation. A fixed, hard in consistency, irregular, nontender, expansive mass of the right mandible was observed on clinical examination. No trismus or other signs of odontogenic infection were mentioned. The panoramic radiograph revealed a mixed radiolucent and radiopaque lesion with ill-defined borders extending from tooth 84 to the mandible angle. A computed tomography scan revealed a suspicious expansive lytic mass of approximately 4.0 × 4.5 cm with cortical erosion and periosteal reaction, suggesting a potential neoplastic process (Fig. 1). Magnetic resonance imaging (MRI) was used to evaluate soft tissue involvement (Fig. 2).

An open incisional biopsy of the lesion was performed, showing a high-density infiltration of small, round, hyperchromatic cells. Immunohistochemical staining with positive and negative controls was also performed, revealing positivity for CD99, CD117, vimentin, BCL2, and epithelial membrane antigen. Antibodies to cytokeratin AE1/3, synaptophysin, desmin, alpha smooth muscle, osteonectin, neuron-specific enolase, CD45, S100, Myo-D1, and CD34 were not reactive with the specimen. Histopathological and immunohistochemical findings were compatible with ES/primitive neuroectodermal tumor (PNET).

**Fig. 3:**
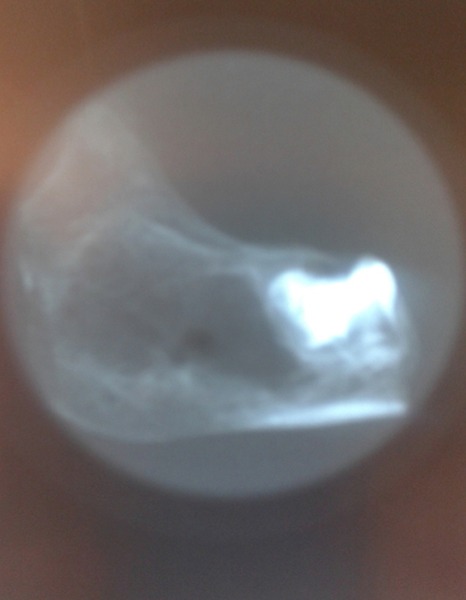
A segmental mandibulectomy, extending from the right mandibular condyle to the tooth 84, was performed

After diagnosis, the patient was referred to the Pediatric Oncology department for further care. Total body bone-scan revealed increased uptake in the right pelvis, which was considered as the primary ES lesion based on tumor’s demographics. The patient was assigned to vincristine, ifosfamide, doxorubicin, and etoposide (VIDE) (EURO-EWING 99) Oncology Protocol, receiving six courses of induction chemotherapy with an almost complete clinical response. A segmental mandibulectomy, extending from the neck of the right mandibular condyle to the distal edge of the right lower primitive 1st molar, was subsequently performed through a submandibular approach (Fig. 3). The uninvolved periosteum was carefully preserved. The tumor was resected with adequate free margins. We decided on delayed reconstruction of the bony continuity defects. The final histopathology report demonstrated only limited tumor residuals, confirming the tumor’s response to the chemotherapeutic regimen. Postoperative chemotherapy was followed in consistency with the oncology protocol.

**Fig. 4:**
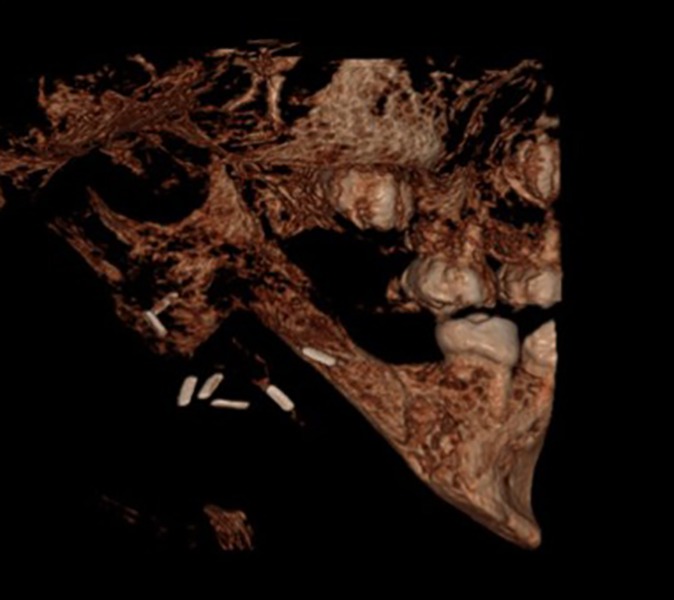
An impresive spontaneous structural and functional regeneration of the resected mandible was reported 2 years after surgery

**Fig. 5:**
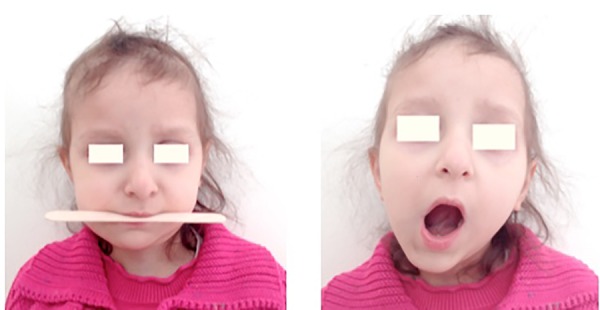
No significant facial asymmetry of functional limitations were observed in a 2-year follow-up evaluation

Clinical and radiological evaluation was performed 2 years after surgery, showing no evidences of locore-gional recurrence. An imprecise reparative reaction of the healthy osseous tissue, leading to an almost complete structural and functional regeneration of the resected mandible, was reported (Fig. 4), making our initial plans for secondary reconstruction unnecessary. There was no significant facial asymmetry, while the patient reported no trismus and regular diet tolerance (Fig. 5). Dental implants, with or without distraction osteogenesis or autogenous bone-grafting techniques, would be used for rehabilitation of the permanent dentition and restoration of normal occlusion. Orthognathic surgery could also be taken under consideration if it was deemed necessary.

## DISCUSSION

A rapidly extensive mass of the mandible in a young-age patient is the most dominant and invariable manifestation of an ES involving the facial bones.^18^ Pain, sensory dysfunctions, loosening teeth, or remitted fever may also be observed in some cases, increasing the suspicion of a malignant process.^9,19-21^ Radiographically, ES could mimic a variety of pathological entities involving the jaws, and thus, it is aptly characterized as the “great imitator of bone pathology” by many authors.^22^ An ill-defined, moth-eaten lytic lesion with or without cortical erosion and bone expansion is the most characteristic radiographic feature,^3,13,23-25^ while the commonly seen long bone periosteal “onion skin” reaction is rarely encountered in jaw lesions.^14-17,26,27^ Computed tomography scans and MRI have been well demonstrated as the most accurate approach for evaluating the extent of the bone destruction and the soft tissue involvement respectively.^3,7,24^ Microscopically, ES is characterized by poorly differentiated, small, round, blue cells.^2,3,28,29^

As ES has similar clinical, radiological, and histo-pathological features to various other malignancies of childhood,^8,10,30-32^ immunohistochemical analysis is crucial for the final diagnosis. Recent genetic studies demonstrated that ES shares a common chromosomal translocation (t:11;22) (q;24:12) with PNETs in more than 90% of cases.^14,18,27,33-35^ The identification of this genetic abnormality, using antibodies to CD99 (Mic-2), is highly sensitive and specific for EW/PNET tumors.^36-40^ Positive staining to vimentin, CD117, BCL2, EMA, FLI1, S-100, glycogen, or pancytokeratin could further support the diagnosis.^7,12,14,24,29,41^

Although the overall prognosis is generally poor due to the tumor’s aggressiveness and the high incidence of early hematogenous dissemination,^2,7,42^ recent multidisciplinary therapeutic protocols and improved chemotherapeutic regimens have significantly improved the 5-year survival from less than 15% to more than 75%.^2,13,43,44^ Combined therapy including wide surgical resection and preoperative and postoperative chemotherapy has been demonstrated as the mainstay of therapeutic approach.^28,45,46^ Radiotherapy is generally avoided as it may induce secondary cancer formation or interfere with facial growth.^47-49^ Vincristine, doxorubicin, cyclophosphamide, ifosfamide, and actinomycin-D are the most widely used anticancer agents for definitive chemotherapy.^50,51^ Demanding function and esthetic reconstruction efforts are usually required for maintaining an acceptable quality of life.

However, this is not the first time that an extensive spontaneous renaissance of a postresection mandible defect has been reported.^52-57^ In contrast with other tissues, bone heals by regeneration rather than scar formation. Patient’s age^54,56,58^ and preservation of peri-osteum^53,54,56,59^ have been described as the main factors that favorably influence this process.

Most cases of spontaneous mandible regeneration, reported in the literature, are in children and young individuals.^52-55,57^ Ihan Hren and Miljavec,^60^ studying the spontaneous healing in 33 patients with large mandible defects, demonstrated that bone-healing capacity was significantly higher (p = 0.006) in patients younger than 20 years. The fact that the cellular activity associated with sequential bone absorption and regeneration is higher in young patients, in conjunction with the presence of abundant mesenchymal cells that can differentiate into osteo-genic cells, offers a satisfactory theoretical background for this statement.^54,56,58^ Periosteum is a well-recognized source of osteoprogenitor cells, and thus, its preservation is crucial in the attempt to maximize the spontaneous regeneration potential.^53,54,56^ Other factors including anatomical location of the defect,^60^ genetic behavior,^52^ and infection^55^ have also been suggested as potential influential factors on mechanism of bone regeneration, but without adequate scientific documentation.

The mandible is the last facial bone that reaches skeletal maturity (14-16 years of age in females and 16-18 years of age in males), and thus, surgical management of jaw pathologic lesions affecting childhood or early adolescence may restrict the mandibular growth, leading to malocclusion and facial asymmetry.^61-63^ It is widely accepted that there are three main growth sites of the mandible. The mandibular condyle is responsible for its vertical growth through endochondral bone formation. Length is gained through constant remodeling of the ramus in response to muscle forces. New bone is deposited at the alveolar process to support teeth development and eruption.^61,64^ Preserving the condylar growth center, as in our case, could, however, minimize the potential of asymmetry or deformity.

## CONCLUSION

The ES/PNET is a highly aggressive malignancy of childhood that rarely affects the bones of the maxillofa-cial region. As it is characterized by undefined clinical, radiological, and histopathological presentation, the immunohistochemical identification of specific chromosomal translocations is the basis of final diagnosis. Current multidisciplinary oncology therapeutic protocols have significantly improved patients’ survival rates, while maintaining high standards of quality of life.

## References

[1] Sanati S, Lu DW, Schmidt E (2007). Cytologic diagnosis of Ewing sarcoma/peripheral neuroectodermal tumor with paired prospective molecular genetic analysis cancer.. Cancer Cytopathol.

[2] Vikas PB, Ahmed MBR, Bastian TS, David TP (2008). Ewing’s sarcoma of the maxilla.. Indian J Dent Res.

[3] Gorospe L, Fernández-Gil MA, García-Raya P, Royo A, López-Barea F, García-Miguel P (2001). Ewing’s sarcoma of the mandible: radio-logic features with emphasis on magnetic appearance.. Oral Surg Oral Med Oral Pathol Oral Radiol Endod.

[4] Ewing J (1921). Diffuse endothelioma of bone.. Proc NY Pathol Soc.

[5] Da Fonseca MA, Abrams RB (1992). Ewing’s sarcoma of the mandible in a young patient: case report.. Pediatr Dent.

[6] Infante-Cossio P, Gutierrez-Perez JL, Garcia-Perla A, Noguer-Mediavilla M, Gavilan-Carrasco F (2005). Primary Ewing’s sarcoma of the maxilla and zygoma: report of a case.. J Oral Maxillofac Surg.

[7] Heare T, Hensley MA, Dell’Orfano S (2009). Bone tumors: osteo-sarcoma and Ewing’s sarcoma.. Curr Opin Pediatr.

[8] Regezi JA, Sciubba J, Jordan PC (2003). Malignancies of the jaws. Oral pathology-clinical pathological correlations..

[9] Wood RE, Nortje CJ, Hesseling P, Grotepass F (1990). Ewing’s tumor of the jaw.. Oral Surg Oral Med Oral Pathol.

[10] Iwamoto Y (2007). Diagnosis and treatment of Ewing’s sarcoma.. Jpn J Clin Oncol.

[11] Langman AW, Kaplan MJ, Matthay K (1989). Ewing’s sarcoma of the mandible.. Otolaryngol Head Neck Surg.

[12] Schultze-Mosgau S, Thorwarth M, Wehrhan F, Holter W, Stachel KD, Grabenbauer G (2005). Ewing sarcoma of the mandible in a child: interdisciplinary treatment concepts and surgical reconstruction.. J Craniofac Surg.

[13] Lopes SL, Almeida SM, Costa AL, Zanardi VA, Cendes F (2007). Imaging findings of Ewing’s sarcoma in the mandible.. J Oral Sci.

[14] Regezi JA, Sciubba J (1999). Oral Pathology..

[15] Behnia H, Motamedi MH (1998). Radiolucent lesion of the man-dibular angle and ramus.. J Oral Maxillofac Surg.

[16] Waldron CA, Neville BW, Damm DD, Allen CM, Bouquot JE (1995). Bone pathology.. Oral and maxillofacial pathology..

[17] Shafer WG, Hine MK, Levy BM (1983). A textbook of oral pathology..

[18] Sai SS, Jambhekar NA (2010). Pathology of Ewing’s sarcoma/PNET: current opinion and emerging concepts.. Indian J Orthop.

[19] Vaccani JP, Forte V, de Jong AL, Taylor G (1999). Ewing’s sarcoma of the head and neck in children.. Int J Pediatr Otorhinolaryngol.

[20] Khanna G, Sato Y, Smith RJ, Bauman NM, Nerad J (2006). Causes of facial swelling in pediatric patients: correlation of clinical and radiologic findings.. Radiographics.

[21] Khoury JD (2005). Ewing sarcoma family of tumors.. Adv Anat Pathol.

[22] Bimal Krishna KB, Thomas V, Kattoor J, Kusumakumari P (2013). A radiological review of Ewing’s sarcoma of mandible: a case report with one-year follow-up.. Int J Clin Pediatr Dent.

[23] Batsakis JG, Mackay B, el-Naggar AK (1996). Ewing’s sarcoma and peripheral primitive neuroectodermal tumor: an interim report.. Ann Otol Rhinol Laryngol.

[24] Yalcin S, Turoglu HT, Ozdamar S, Sadikoglu Y, Gurbuzer B, Yenici O (1993). Ewing’s tumor of the mandible.. Oral Surg Oral Med Oral Pathol.

[25] Mubeen AS, Telkar S (2007). Ewing’s sarcoma of the ramus of mandible: report of a case.. J Indian Assoc Oral Med Radiol.

[26] Ward-Booth P, Peterson LJ, Indresano AT, Marciani RD, Roser SM (1992). Surgical management of malignant tumors of the jaws and oral cavities.. Principles of oral and max-illofacial surgery, Chapter 32. Vol. 2..

[27] Krane MS, Schiller AL, Isselbacher KJ, Braunwald E, Wilson JD, Martin JB, Fauci AS, Kasper DL (1994). Hyperostosis, neoplasms, and other disorders of bone and cartilage.. Harrison’s principles of internal medicine, Chapter 326. Vol. 2..

[28] Fonseca AS, Mezzalira R, Crespo AN, Bortoleto AE Jr, Paschoal JR (2000). Ewing’s sarcoma of the head and neck.. Sao Paulo Med J.

[29] Talesb KT, Motamedi MH, Jeihounian M (2003). Ewing’s sarcoma of the mandibular condyle: report of a case.. J Oral Maxillofac Surg.

[30] Brazão-Silva MT, Fernandes AV, Faria PR, Cardoso SV, Loyola AM (2010). Ewing’s sarcoma of the mandible in a young child.. Braz Dent J.

[31] Brinkhuis M, Wijnaendts L, Van der Linden J (1995). Peripheral primitive neuroectodermal tumor and extraosseus ES: a histo-logical, immunohistochemical and DNA flow cytometric study.. Virchows Arch A Pathol Anat Histopathol.

[32] Ozer E, Kanlikama M, Karakurum G, Sirikci A, Erkilic S, Aydin A (2002). Primitive neuroectodermal tumor of the mandible.. Int J Pediatr Otorhinolaryngol.

[33] Kapadia SB, Barnes L (2001). Tumors of the nervous system.. Surgical pathology of the head and neck..

[34] Dorfman HD, Czerniak B (1998). Ewing’s sarcoma and related entities. Bone tumors..

[35] German J, Isselbacher KJ, Braunwald E, Wilson JD, Martin JB, Fauci AS, Kasper DL (1994). Cytogenic aspects of human disease.. Harrison’s principles of internal medicine, Chapter 62. Vol. 2..

[36] Sorensen P, Shimada H, Liu XF (1995). Biophenotypic sarcomas with myogenic and neural differentiation express the Ewing’s sarcoma EWS/FLI1 fusion gene.. Cancer Res.

[37] Perlman EJ, Dickman PS, Askin FB, Grier HE, Miser JS, Link MP (1994). Ewing’s sarcoma-routine diagnostic utilization of MIC2 analysis: a Pediatric Oncology Group/Children’s Cancer Group Intergroup Study.. Hum Pathol.

[38] Weidner N, Tjoe J (1994). Immunohistochemical profile of monoclonal antibody O13: antibody that recognizes glycoprotein p30/32MIC2 and is useful in diagnosing Ewing’s sarcoma and peripheral neuroepithelioma.. Am J Surg Pathol.

[39] Sandberg AA, Bridge JA (2000). Updates on cytogenetics and molecular genetics of bone and soft tissue tumors: Ewing sarcoma and peripheral primitive neuroectodermal tumors.. Cancer Genet Cytogenet.

[40] Vicha A, Stejskalova E, Sumerauer D, Kodet R, Malis J, Kucerova H, Bedrnicek J, Koutecky J, Eckschlager T (2002). Malignant peripheral primitive neuroectodermal tumor of the kidney.. Cancer Genet Cytogenet.

[41] Weiss SW, Goldblum JR (2001). Soft tissue tumor and Ewing’s sarcoma histologic and immunohistochemical staining (MIC 2)..

[42] Berk R, Heller A, Heller D, Schwartz S, Klein EA (1995). Ewing’s sarcoma of the mandible: a case report.. Oral Surg Oral Med Oral Pathol Oral Radiol Endod.

[43] Ozaki T, Hillmann A, Hoffmann C, Rube C, Blasius S, Dunst J, Jurgens H, Winkelmann W (1996). Significance of surgical margin on the prognosis of patients with Ewing’s sarcoma. A report from the Cooperative Ewing’s Sarcoma Study.. Cancer.

[44] Wexler LH, DeLaney TF, Tsokos M, Avila N, Steinberg SM, Weaver-McClure L, Jacobson J, Jarosinski P, Hijazi YM, Balis FM (1996). Ifosfamide and etoposide plus vincristine, doxorubicin, and cyclophosphamide for newly diagnosed Ewing’s sarcoma family of tumors.. Cancer.

[45] Bernstein M, Kovar H, Paulussen M, Randall RL, Schuck A, Teot LA, Juergens H (2006). Ewing’s sarcoma family of tumors: current management.. Oncologist.

[46] Van der Woude HJ, Bloem JL, Hogendoorn PC (1998). Preoperative evaluation and monitoring chemotherapy in patients with high-grade osteogenic and Ewing’s sarcoma: review of current imaging modalities.. Skeletal Radiol.

[47] Kuttesch JF Jr, Wexler LH, Marcus RB, Fairclough D, Weaver-McClure L, White M, Mao L, Delaney TF, Pratt CB, Horowitz ME (1996). Second malignancies after Ewing’s sarcoma: radiation dose-dependency of secondary sarcomas.. J Clin Oncol.

[48] Votta JV, Fantuzzo JJ, Boyd CB (2005). Peripheral primitive neuroec-todermal tumor associated with the anterior mandible: a case report and review of the literature.. Oral Surg Oral Med Oral Pathol Oral Radiol Endod.

[49] Koscielniak E, Morgan M, Treuner J (2002). Soft tissue sarcoma in children: prognosis and management.. Paediatr Drugs.

[50] Sharada P, Girish HC, Umadevi HS, Priya NS (2006). Ewing’s sarcoma of the mandible.. J Oral Maxillofac Pathol.

[51] Gosau M, Baumhoer D, Ihrler S, Kleinheinz J, Driemel O (2008). Ewing sarcoma of the mandible mimicking an odontogenic abscess-a case report.. Head Face Med.

[52] Ogunlewe OM, Akinwande AJ, Ladeinde LA, Adeyemo LW (2006). Spontaneous regeneration of whole mandible after total mandibulectomy in a sickle cell patient.. J Oral Maxillofac Surg.

[53] Adekeye EO (1997). Rapid bone regeneration subsequent to subtotal mandibulectomy. Report of an unusual case.. Oral Surg Oral Med Oral Pathol.

[54] Park HW, Kim HJ, Park BM (1997). Spontaneous regeneration of the lateral malleolus after traumatic loss in a three-year-old boy: a case report with seven-year follow-up.. J Bone Joint Surg Br.

[55] Whitmyer CC, Esposito SJ, Smith JD, Zins JE (1996). Spontaneous regeneration of a resected mandible in a preadolescent. A clinical report.. J Prosthet Dent.

[56] Ruggiero SL, Donoff RB (1991). Bone regeneration after mandibular resection: report of two cases.. J Oral Maxillofac Surg.

[57] Nagase M, Ueda K, Suzuki I, Nakajima T (1985). Spontaneous regeneration of condyle following hemimandibulectomy by disarticulation.. J Oral Maxillofac Surg.

[58] Arrington ED, Smith WJ, Chambers HG, Bucknell AL, Davino NA (1996). Complications of iliac crest bone graft harvesting.. Clin Orthop Relat Res.

[59] Lemperle SM, Calhoun CJ, Curran RW, Holmes RE (1998). Bony healing of large cranial and mandibular defects protected from soft-tissue interposition: a comparative study of spontaneous bone regeneration, osteoconduction, and cancellous autograft- ing in dogs.. Plast Reconstr Surg.

[60] Ihan Hren M, Miljavec M (2008). Spontaneous bone healing of the large bone defects in the mandible.. Int J Oral Maxillofac Surg.

[61] Smartt JM Jr, Low DW, Bartlett SP (2005). The pediatric mandible: I. A primer on growth and development.. Plast Reconstr Surg.

[62] Farkas LG, Posnick JC, Hreczko T (1992). Anthropometric growth study of the head.. Cleft Palate Craniofac J.

[63] Moss ML, Salentijn L (1969). The primary role of functional matrices in facial growth.. Am J Orthod.

[64] Posnick J (2014). Orthognathic surgery: principles and practice..

